# Bayes Factor vs. Posterior Predictive Model Assessment: Insights from Ordinal Constraints

**DOI:** 10.1007/s42113-025-00240-0

**Published:** 2025-04-02

**Authors:** Julia M. Haaf, Fayette Klaassen, Jeffrey N. Rouder

**Affiliations:** 1 Department of Psychology, University of Potsdam, Potsdam, Germany; 2 Amsterdam Institute for Global Health and Development, Amsterdam, the Netherlands; 3 University of California, Irvine, CA USA

**Keywords:** Bayesian inference, Watanabe-Akaike information criterion, Bayes factor, Ordinal-constrained inference

## Abstract

A central element of statistical inference is a good model specification where researchers specify models that capture differing theoretical positions. We argue that methods of inference forcing researchers to use models that may not be appropriate for their research question are not as desirable as methods with no such constraints. We ask how posterior predictive model assessment methods such as Watanabe–Akaike information criterion and leave-one-out cross-validation perform when theoretical positions correspond to different space restrictions on a common parameter space. One of the main theoretical relations is nesting—where the parameter space of one model is a subset of that for another. A good example is a general model that admits any set of preferences; a nested model is one that admits only preferences that obey transitivity. We find that posterior predictive methods fail in these cases: More constrained models are not favored even when data are compatible with the constraint. Researchers who use posterior predictive methods are forced to partition the parameter space into non-overlapping subspaces, even if these subspaces have no theoretical interpretation. Fortunately, Bayes factor model comparison accommodates overlapping models without such difficulties. We argue given that posterior predictive approaches force certain specifications that may not be ideal for scientific questions, they are less desirable in many substantive applications.

One pillar of science is that data inform substantive statements about constraints in the world. Exactly how to do this, that is, how to draw inferences, remains perennially contested. In this paper, we ascribe to the popular view of *model comparison.* Accordingly, theoretical positions are represented as statistical models which are then compared with one another on grounds of fit and parsimony. And just as inference remains perennially contested, so do methods of model comparison. We contrast two Bayesian model comparison methods here: Bayes factor (Jeffreys, 1961; Kass & Raftery, 1995) and posterior predictive information criteria (Vehtari et al., 2017).

One element of inference that receives relatively little attention is the importance of good model specification. In Bayesian statistics, model specification includes the specification of the full likelihood and prior distributions for all parameters. We think good model specification produces tractable models that reflect the theoretical questions under consideration. While there may be little doubt that good model specification is sensible, this element of inference likely receives less attention for a number of reasons including that good specification requires substantive knowledge. Therefore, whether a specification is good or bad will vary from context to context and field to field, and that makes global pronouncements limited in value.

What is not limited in value is the following perspective from Singmann et al. (2023). These authors worry that researchers analyze their scientific questions with readily available, default statistical models rather than tailoring the model to the scientific question at hand. We call this perspective—that model specification is primary and, by extension, that statistical inference is secondary for scientific inference—the *specification-first* principle. If we take specification as primary, then it follows that good model comparison methods should allow researchers to specify whatever models best fit their scientific questions at hand and still yield reasonable inferences.

In this paper, we use the specification-first principle as a test to highlight a weakness of posterior predictive methods and a strength of Bayes factors. We are certainly not the first to compare these methods—the literature about various methods of Bayesian inference is vast, and we have no desire to rehash the debate. Readers interested in the debate are referred to Chandramouli and Shiffrin (2019); Gronau and Wagenmakers (2019); Gelman and Shalizi (2013); Morey et al. (2016); Navarro (2019); Wagenmakers (2007); Vanpaemel (2010) among many others. Here, our focus is on the logical ramifications of honoring the specification-first principle. We argue that model comparison by the Bayes factor honors the specification-first principle while model comparison by posterior predictive methods does not.

The case we consider is where one model, a constrained model, is nested in a more general unconstrained model. For example, an unconstrained model may be specified by requiring a certain parameter, denoted $\theta$, which has full support of the reals, $-\infty<\theta <\infty$. A constrained model may require that this parameter has the support of the positive reals, $0<\theta <\infty$. Note that there are collections of parameter values, in this case, the positive ones, that are supported in both the unconstrained and constrained models—the models overlap. In the normal course of things, when data are compatible with both models, the constrained model should fare better in model comparison because it predicted the data could be within the constraint and not anywhere else. The unconstrained model made no such upfront prediction; hence, it is not preferred by Occam’s razor (de Finetti, 1974). Science at its root is about understanding constraints on the natural world, and reasonable methods of inference should be able to state evidence for constraints when they hold.

Herein lies the problem for posterior predictive methods. If the data are highly compatible with the constraint, posteriors under both the unconstrained and constrained models are approximately the same. Any prediction from them is the same, and posterior predictive methods would provide an inference that both the unconstrained model and the constrained model are equivocal. Scientists are unable to draw inferences for constraints or simplifications in this case.

This problem with posterior predictive inference is illustrated in three examples. In each, we derive the Watanabe–Akaike information criterion (WAIC) as an example method as well as the Bayes factor. We show that WAIC gives the wrong inferential insight. In the first example, with order constraint on one parameter, it is clear that WAIC is able to adjudicate between models with certain specifications and not others whereas there is no such distinction for the Bayes factor. This example is the simplest, but it is also the most controversial as one could conceivably argue the statistical advantages of the models for which WAIC works may outweigh substantive concerns. The subsequent two examples, though more complicated and narrower in scope, are more convincing. Here, the models that WAIC can distinguish are untenable from a substantive point of view as they violate the independence of participants (Example 2) or make the results substantively uninterpretable (Example 3). It is the sum total across the three examples that make our concluding point—Bayes factors are useful for assessing ordinal constraints, a class of constraints that is particularly useful for scientific inquiry in psychological science (Haaf et al., 2019; Ludwig et al., 2023), and posterior predictive methods are not.

## Example 1: A Randomized Clinical Trial

As implausible as it sounds, there is a certain stable genius who happens to be the bestest real-estate businessman, politician, general, epidemiologist, and virologist. And this stable genius has recommended that the drug hydroxychloroquine be used both to treat and prevent Covid-19 infections. Yet, the leading American pandemic expert, Dr. Fauci, is not convinced without data. And so, the stable genius and Dr. Fauci decide to run an experiment in an adversarial collaboration. Suppose confirmed Covid-19 sufferers are randomly assigned to treatment (hydroxychloroquine) and control (placebo tablet) groups. The outcome is the change in severity of symptoms after a set time, say 96 h. To make it interesting, Dr. Fauci and the stable genius bet a case of medical-grade N95 masks. We happen to be called in as statisticians to determine who wins in light of the data.

Let $Y_{ij}$ be the symptom change measure for the $j$th person in the $i$th group, where $i=0$ for the control group and $i=1$ for the hydroxychloroquine group. We start with the following specification:

$$\begin{aligned} Y_{ij} \sim \text {Normal}(\mu -x_i\theta ,\sigma ^2), \end{aligned}$$

where $\mu$ is an overall symptom change, $x_i=0,1$ for the control and hydroxychloroquine conditions, respectively, and $\theta$ is the reduction in symptoms from the hydroxychloroquine conditions relative to control condition.

The stable genius’ theory can be reflected with the constraint $\theta >0$. But what should be the constraint for Dr. Fauci’s model? The usual null constraint is $\theta =0$, but Dr. Fauci is concerned that this drug could possibly exacerbate Covid-19 symptoms. So perhaps Dr. Fauci should stake out the constraint that $\theta <0$. But, then again, Dr. Fauci is not truly committed to this direction. Perhaps he would prefer to not place a constraint on $\theta$.

One advantage of Bayesian model specification is its flexibility. All of the above constraints and the lack of a constraint are easily encoded into formal models through the specification of the prior. The stable genius specifies $\theta \sim \text {N}_+(0,b^2)$, where $N_+$ is the normal distribution truncated below at zero and $b^2$ is prior variance setting. Dr. Fauci considers three additional specifications. The first is the null model, $\theta =0$. The second is the negative model, $\theta \sim \text {N}_-(0,b^2)$, where $N_-$ is the normal distribution truncated above at zero. The third model is the unconstrained model, $\theta \sim \text {Normal}(0,b^2)$.

Which of Dr. Fauci’s three models is the best? Here is where the specification-first principle comes into play. The choice of best is a substantive rather than a statistical question. All three are valid models, and by the specification-first principle, the choice between them should reflect substantive rather than inferential concerns. As an aside, our tendency in cases like this would be to choose the unconstrained model; this model reflects a fair appraisal of Dr. Fauci’s open-mindedness. Unconstrained models have another advantage. They allow researchers to detect unexpected events, say a dramatic negative effect of hydroxychloroquine on symptom relief; hence, they serve as a guard against model misspecification.

**Fig. 1:**
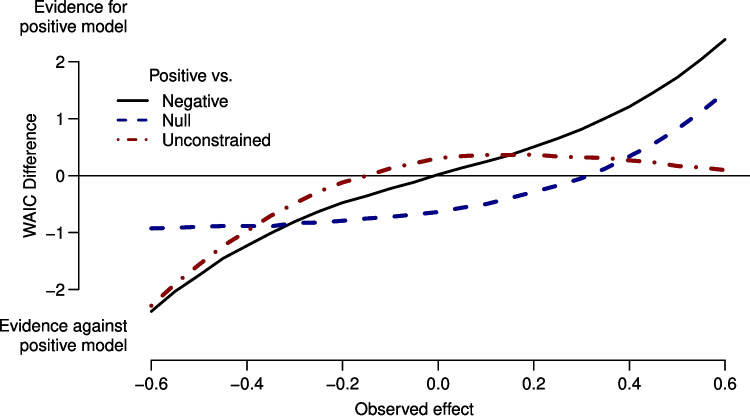
Model comparisons with WAIC for the hydroxychloroquine study. The positive model is compared to the three possible choices for Dr. Fauci. Positive effects are in line with the positive model, and a positive WAIC difference corresponds to evidence for the positive model

Suppose we choose to use model comparison by WAIC (Vehtari et al., 2017), an increasingly popular Bayesian model comparison criterion that stresses how well models predict out-of-sample data. Figure 1 displays the WAIC comparison statistic for this case as a function of the size of the difference between treatment and control conditions. The comparison is the model of the stable genius, the positive model where $\theta >0$, against each of the three models that Dr. Fauci might choose. The solid line is for the positive model vs. the negative model. The behavior here is desirable and intuitive—larger positive observed effects correspond to relatively more evidence for the stable genius. The dashed line is the comparison between the positive model and the null model. Again, the behavior makes sense. Large positive observed effects provide evidence for the positive model.

Where WAIC becomes undesirable and counter-intuitive is when Dr. Fauci chooses the unconstrained model (the dashed-dotted line). Model comparison for large negative observed effects makes sense—as the effect becomes more negative there is increasing evidence for Dr. Fauci’s position. Yet, the same is not true for increasingly large positive values. One would think that as the values become increasingly positive there should be increasing evidence for the stable genius’ position. Yet, the opposite occurs, there is less evidence, and in the limit of large observed effects, the models are equivalent according to WAIC (see Fig. 1). Even though the stable genius made a more precise prediction than Dr. Fauci, and one that is born out in the data, he can gain no advantage.

What is demonstrated in this example is that WAIC is not appropriate for all model comparisons. It certainly is appropriate for the positive-vs-negative models and positive-vs-null, but is inappropriate for the positive-vs-unconstrained model. It places limits on the positions that substantive scientists may take, and in this sense, violates the specification-first principle.

Why does this undesirable behavior occur for WAIC with certain models? In the next section, we review WAIC and show that posterior predictive inference is the source of the problem. Posterior predictions may converge to the same value when models overlap, even though one model may be more constrained than the other. This issue also applies to other posterior predictive methods, including the deviance information criterion (DIC, Spiegelhalter et al., 2002) and leave-one-out cross-validation (LOO, Vehtari et al., 2017). In the section after, we review the Bayes factor. Because the Bayes factor relies on prior information to make predictions, space constraints are properly accounted for even with model overlap.

## Model Comparison with WAIC

Posterior predictive methods start by making a *prediction* about new data given a current set of data and a model. Say, for example, the model is $y_1,\ldots ,y_n \sim \text {M}(\theta )$ subject to a prior $\pi (\theta )$ on parameters $\theta$.^1^ We then ask what is the prediction for the next observation $y^*$. In this case, the prediction is denoted $p(y^* \mid y_1,\ldots ,y_n)$ and is expanded as

$$ p(y^* \mid y_1,\ldots ,y_n) = \int _\theta p(y^*\mid \theta )p(\theta \mid y_1,\ldots ,y_n)d\theta . $$

If we had a new data set of independent observations, say $y^*_1,\ldots ,y^*_m$, then we could competitively see how well different models predicted these values from the original set by computing $\sum _i^m \log p(y^*_i \mid y_1,\ldots ,y_n)$. Gelman et al. (2014) call this quantity the *log point-wise predictive distribution* (lppd).

Here is an example. Suppose $y_1,\ldots ,y_n \sim \text {N}(\mu ,1)$ subject to the prior $\mu \sim \text {N}(0,b)$. The posterior distribution is $\mu \mid y_1,\ldots ,y_n \sim \text {N}(cv,v)$, where $c=n \bar{y}$ and $v=(n+1/b)^{-1}$. With this, we note that $p(y^* \mid y_1,\ldots ,y_n) \sim \text {N}(cv,v+1)$, and, if we had many new observations, $y^*_1,\ldots ,y^*_m$, then

$$\begin{aligned} \text {lppd} =&\sum _i^m \log p(y^*_i \mid y_1,\ldots ,y_n)\\ =&\sum _i^m \left( -\frac{1}{2}\log (2\pi ) -\frac{1}{2}\log (1+v)-\frac{1}{2(1+v)}(y^*_i-c(1+v))^2\ \right) \end{aligned}$$

The usual practice is to use the original data as the new data. The notation has to change a bit because if we continue, the density $p(y_i \mid y_1,\ldots ,y_n)$ seems degenerate. Here, to emphasize that we are not using each data point to predict itself, but that predictions are made with reference to the posterior, we are going to use the notation, $p^*(y_i|y_1,\ldots ,y_n)$ to indicate the posterior predictive distribution of the observed data. For the normal model with unknown mean, the lppd becomes the following:

$$\begin{aligned} \text {lppd}&= \sum _i^n \log p^*(y_i \mid y_1,\ldots ,y_n)\\&= \sum _i^n \left( -\frac{1}{2}\log (2\pi ) -\frac{1}{2}\log (1+v)-\frac{1}{2(1+v)}(y_i-c(1+v))^2\ \right) \end{aligned}$$

Of course, using the existing data that is already used for the posterior distribution adds bias. To correct for this bias, the usual approach is to make an *effective number of parameters* correction. Computing these corrections is technical, and for the purposes herein, we use the recommended WAIC-2 correction of Gelman et al. (2014). This correction, denoted $p$, is based on the variability of estimates.

$$ p = \sum _i^n V_{\theta |y_1,\ldots ,y_n}\log p^*(y_i \mid y_1,\ldots ,y_n), $$

Here, the variance is taken with respect to the posterior distribution. Gelman et al. (2014) provides an expression for MCMC chains.

**Fig. 2:**
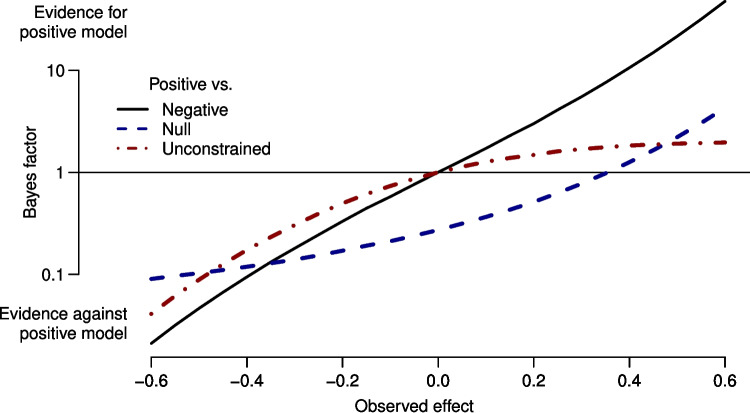
Model comparisons with Bayes factor for the hydroxychloroquine study. The positive model is compared to the three possible choices for Dr. Fauci. Positive effects are in line with the positive model, and a Bayes factor above 1 corresponds to evidence for the positive model

## Posterior Predictive Methods Depend on the Posterior Distribution

One of the important properties of both the lppd prediction term and the correction is that each is conditional on the posterior predictive distribution. In case of the lppd, we take the sum of the log posterior predictive distribution for each observation. In case of the correction term, $p$, we take the sum of the variance of the log predictive distribution for each observation. The posterior predictive distribution is based on the posterior distribution of the parameters given the data. Hence, if two models yield the same posterior, then the WAIC will be the same. And here, we see the source of the problem in Fig. 1. Say the hydroxychloroquine trials were wildly successful. The posterior under the positive model and the unconstrained model are just about the same. Therefore, the WAIC model comparison statistics are just about the same. Even though the stable genius proposed a testable hypothesis, and even if he wins bigly, he cannot beat Dr. Fauci’s unconstrained model with WAIC.

The WAIC is not the only information criterion that fails to distinguish between models with similar posterior distributions. Any model comparison technique relying on posterior prediction faces the same issue, including DIC, which is based on the expected value of the posterior distribution, and LOO-CV, which is based on out-of-sample posterior prediction where the hold-out data consists of one observation. All of these methods use posterior prediction to penalize for model complexity. If the posterior distributions of two models are similar, then the penalty term is also similar. To settle the bet using posterior predictive methods, we are forced to assign to Dr. Fauci the view that hydroxychloroquine has a negative effect or a zero effect, a view he may not hold, and certainly a violation of the specification-first principle.

## Model Comparison with Bayes Factor

Figure 2 shows the same model comparisons with the Bayes factor rather than with WAIC (again, the computations are for known variance and unknown mean). Again, when Dr. Fauci commits to the negative model, the Bayes factor comparison with the positive model seems straightforward—larger sample effects provide relatively more evidence for the positive model. The comparison of the null model to the positive model has this monotonicity as well. The critical comparison is between the unconstrained and the positive model. Here, the curve looks similar to the previous one with WAIC, but with one critical difference. The Bayes factor asymptotes to 2-to-1 in favor of the stable genius as the sample mean difference increases. This difference may look small, but it is quite consequential.

The factor of 2-to-1 reflects the underlying complexities of the models. The positive model has a parameter space that is half of the parameter space of the unconstrained model. Hence, if the data fall definitively within the positive space, it was predicted by a factor of 2-to-1 in favor of the positive model. This factor reflects parsimony, or the degree of risk, which is why the Bayes factor is considered to have a natural penalty for model complexity (Myung & Pitt, 1997).

At this point, an analyst might be unsettled by the unconstrained model on statistical grounds. The WAIC is inappropriate, and the BF is limited in the strength of evidence shown for the constraint. An intrepid analyst might be tempted to specify models that do not overlap, or at least not overlap significantly. In this example, the analyst might advise Dr. Fauci to take the *complement* of the stable genius’ position, that is, the negative values. In this case, model comparison between the negative and positive models is appealing on statistical grounds as BF and WAIC comparison statistics grow without bounds as the data become more extreme. Such an approach might make sense, but it is a violation of the specification-first principle if Dr. Fauci prefers the unconstrained model on substantive grounds. We call the intrepid analyst’s position the *no-overlap approach* as there is no overlap in the support of $\theta$ across the models. Here, statistical considerations are used to justify models without overlap. Shiffrin et al. (2016) and Chandramouli and Shiffrin (2019) provide detailed discussions about such statistical considerations motivating a no-overlap approach. In subsequent examples, we take this no-overlap approach seriously. We show in common cases it results in models that have troubling substantive implications. If substantive concerns are primary, then models should be allowed to overlap, and posterior predictive methods are inappropriate.

It may seem problematic that there is a 2-to-1 upper limit on the amount of evidence the stable genius can state for the effectiveness of hydroxychloroquine. Yet, it is a natural consequence that the stable genius did not specify that much constraint. The stable genius could make riskier predictions in an enhanced design. Here is an example: Suppose the stable genius and Dr. Fauci now agree to run a three-site study. The main parameters are symptom change for the placebo group, denoted $\mu _{0j}$, $j=1,2,3$ for the three sites, and symptom change for the hydroxychloroquine group, denoted $\mu _{1j}$, $j=1,2,3$. The stable genius has now imposed three constraints, $\mu _{1j}>\mu _{0j}$ for the three sites. Let us suppose Dr. Fauci remains uncommitted and the data come back with positive sample mean differences at all three sites. With WAIC, we run into the same problem. The posteriors under the uncommitted model and positive model are similar, and the WAIC is equivalent. With Bayes factors, however, the valid parameter space is cut down by 8 (a factor of 2 for each constraint), and the resulting Bayes factor asymptotes at 8-to-1 in favor of the stable genius.^2^ The Bayes factor is appropriately factoring in the constraints.

## Example 2: Independence of Individuals

Researchers in cognition often consider hierarchical data where trials are nested in individuals. Let us consider a priming task in which several individuals run several trials each in a primed and unprimed condition. One of the critical substantive requirements of specification is the preservation of the independence of individuals. Usually, this requirement is trivial to satisfy, but we show here that there is an incompatibility between posterior predictive methods and independence across participants.

As substantive scientists, we often know a priming effect exists in the aggregate. For example, on average, people show a Stroop effect. The question we tend to ask in our own work is about the collective of individuals—do *all* individuals show a true priming effect? If a priming effect holds for all individuals, then we may think that priming is automatic and outside of volitional control (Haaf & Rouder, 2017). Conversely, if the priming effect holds only for some but not all people, we may think there may be a strategic component.

Let $Y_{ijk}$ denote the $k$th replicate response time for the $i$th individual in the $j$th condition:

$$ Y_{ijk} \sim \text {N}(\mu _i+x_j\theta _i,\sigma ^2), $$

where $\mu _i$ is the overall speed for the $i$th individual, $x_j=-.5,.5$ indicates the priming condition, $\theta _i$ is the priming effect for the $i$th individual, and $\sigma ^2$ is trial-to-trial variability. The *everybody does* model is specified by placing the following prior on $\theta _i$: $\theta _i|\nu ,\delta ^2 \overset{iid}{\sim }\ \text {N}_+(\nu ,\delta ^2)$. We see here that (a) individuals have true positive effects and (b) conditional on population parameters $\nu$ and $\delta ^2$, and individual true values are independent of one another.

What model could we use to express a violation of the everyone does model? One choice is an unconstrained model: $\theta _i|\nu ,\delta ^2 \overset{iid}{\sim }\ \text {N}(\nu ,\delta ^2)$. Here, individuals may have positive or negative true values, and, importantly, conditional independence is preserved. Another possibility is the complement where at least one person has a negative true value. We think the complement model, however, is quite difficult *from a substantive perspective* because it violates conditional independence. If one knows that $I-1$ individuals have a positive priming effect, then one knows that the remaining individual must have a negative priming effect! This violation of independence makes no sense in our experiments where participants are run separately. And so, the adoption of the complement model is a violation of the specification-first principle.

**Fig. 3:**
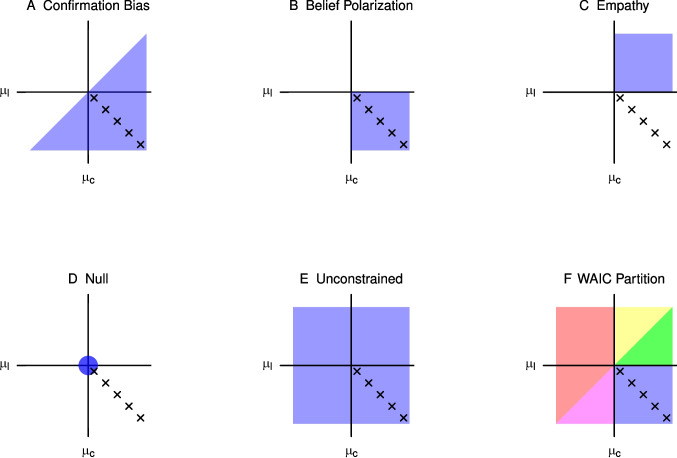
The first five plots show models motivated by substantive considerations. The last plot is the partition of the parameter space necessitated by posterior predictive model comparisons. The Xs represent example sample means concordant with the belief-polarization model

## Example 3: Substantive Theory of Belief Polarization

In this section, we provide a hypothetical that better interfaces with common examples in the social sciences. Suppose we have developed a documentary that explores the plight of Central American migrants seeking asylum in the United States. The documentary examines the causes of their migration, the burdens they have faced, and their aspirations in migrating. And it is designed to induce a more favorable response toward these migrants. To assess the effects of the documentary, attitudes toward migrants are assessed before and after its viewing. There are two specific substantive theories from the literature the researchers wish to assess:

### Confirmation Bias

This position is informed by classic work by Tversky and Kahneman (1974) on confirmation bias. People may ignore media stories that are incompatible with their worldview. In the current political climate, many conservatives endorse a law-and-order view where Central American migrants may present a direct threat to their welfare and security. Hence, some conservatives may ignore or downweigh media stories sympathetic to these migrants. Accordingly, liberals should be more positively affected by these stories than conservatives.

### Belief Polarization

This position is motivated by more recent work of Cook and Lewandowsky (2016) who studied the effects of presenting scientific information on beliefs about global climate change. They document a phenomenon called *belief-polarization*. When presented with this scientific information, liberals and conservatives had markedly different responses. The information led liberals to believe more strongly in climate change and led conservatives to disbelieve more strongly in it. The increased disbelief of conservatives when presented with valid data evidencing climate change is paradoxical. Their attitudes are moving in the opposite direction of the information. Cook and Lewandowsky show that this phenomenon may be explained by considering how people treat the validity of information they do not agree with. In this case, the conservative respondents see the scientific information as fraudulent, and the fact that researchers must resort to supposed fraudulent information to support global warming is viewed as further evidence against its existence. Could the same belief-polarization dynamics be present for the migration documentary? Accordingly, liberals might see refugees more favorably after viewing positive stories while conservatives might actually see them more negatively.

**Fig. 4:**
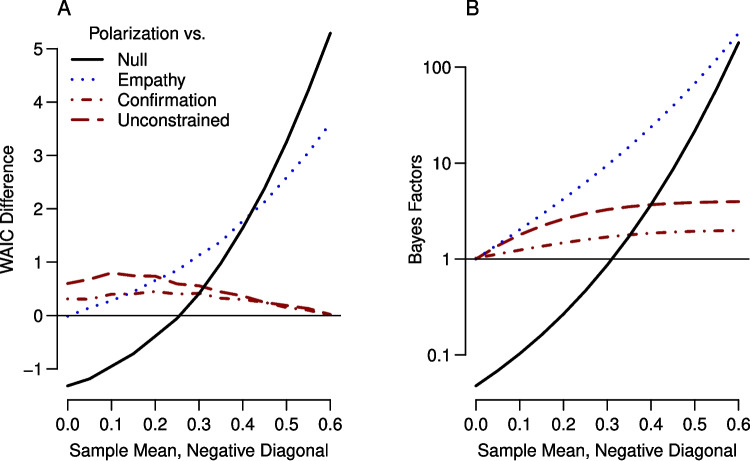
Model comparison for sample means compatible with the belief-polarization model. The lines show the comparison of this model with the competitors. **A** Model comparison by WAIC. **B** Model comparison by Bayes factor

### Empathy

Suppose the change in favorability does not depend on political affiliation per se. Instead, these changes depend on how empathetic the viewer is. Social empathy increases with proximity and familiarity (Segal, 2011). Therefore, whenever we learn more about the situation of a social group, empathy should increase. Whereas we are unsure whether self-identified liberals or conservatives are more empathetic on balance, we implement this model by requiring that the mean change be positive for liberals and conservatives alike.

In addition to these three models, we should carry two models that reflect a degree of humbleness about our instrument and theories. The first is that the documentary is ineffective. We can call this the **null model**, and, accordingly, there is no effect on attitudes for liberals or conservatives. Second, we should note that our substantively inspired models do not cover the space of all possibilities. They could all be wrong. To account for this misspecification, we include an **unconstrained model** where we make no commitments to the orderings of mean change in attitudes. A sketch of the five models is shown in Fig. 3 A–E.

We are now ready to instantiate these positions as models. Let $Y_i$, $i=1,\ldots ,I$ denote the change in favorability for the $i$th participant who viewed the documentary. For the purposes herein, we are going to model this change as a continuous variable as

$$ Y_i \overset{ind}{\sim }\text {Normal}(x_i\mu _\ell +[1-x_i]\mu _c,\sigma ^2), $$

where $x_i$ is an indicator which is 1 if the $i$th person is liberal and 0 if the $i$th person is conservative. The parameters of interest are $\mu _\ell$ and $\mu _c$, and the true attitude changes for liberals and conservatives, respectively.

The models are implemented as follows. We place a prior $(\mu _c,\mu _\ell )' \sim N_2(\boldsymbol{0},b\boldsymbol{I})$, where $N_2$ is a bivariate normal, $\boldsymbol{I}$ is the 2-by-2 identity matrix, and $b$ is a scale setting. For the confirmation bias model, we add the constraint that $\mu _c<\mu _\ell$. For the belief-polarization model, we add the constraint $\mu _c<0<\mu _\ell$. For the empathy model, we add the constraint that $\mu _c>0$ and $\mu _\ell >0$. For the null model, we add the constraint $0=\mu _c=\mu _\ell$. Finally, for the unconstrained model, we add no additional constraints to the prior.

To explore the critical behavior of model comparison methods, we evaluated them for sample means concordant with the belief-polarization model. These sample means, along the negative diagonal, are shown as Xs in Fig. 3. The resultant behavior of WAIC and Bayes factors is shown in Fig. 4. The plots show the comparison of the belief-polarization model vs. all others, and as we progress further down the negative diagonal, the comparisons should provide greater support for this model.

For the Bayes factor, the belief polarization gains increasing support against all other models. The asymptotes of the unconstrained and the confirmation bias model reflect the size of the constraint. The parameter space of the unconstrained model is four times as large as that of the belief-polarization model. Therefore, the Bayes factor asymptotes to 4.0; likewise, because the confirmation model has twice the parameter space than the belief-polarization model, the Bayes factor asymptotes to 2.0.

For WAIC, the belief-polarization model is indeed better supported than the null and empathy models. Yet, again, there is the undesirable result where belief polarization, confirmation bias, and the unconstrained model receive the same degree of support as we progress down the negative diagonal. Even though the belief-polarization model entails detailed, substantively motivated constraints, and even though the data are squarely compatible with it, this model receives no greater support than the unconstrained model. This, in our view, is problematic for theory assessment.

An intrepid analyst who wished to use WAIC might partition the parameter space exhaustively without overlap. One possible partitioning result is shown in Fig. 3F. We would allot the lower right quadrant to the belief-polarization model, the pink and the green triangle to the confirmation bias model, the yellow triangle to the empathy model, and, finally, the awkward red area consisting of the red triangle (lower left) and the upper left quadrant to the unconstrained model. Using this partitioning, only the area allotted to the belief-polarization model is in accordance with its theoretical content. We could apply WAIC, but we are at a loss as of how to interpret the results. The gravest issue is that we could also decide to allot these partitions differently: How about assigning the lower right quadrant and the pink triangle to the confirmation bias model, the green triangle and the red area to the unconstrained model and the yellow triangle stays with the empathy model? In this assignment, the belief-polarization model misses out, but the theoretical justification for this assignment is as good as for the first one.

## Discussion

In this paper, we advocate that model comparison methods should adhere to the specification-first principle: The models specified should be a judicious representation of theoretical considerations and not a function of the limitations of model comparison methods. Using ordinal-constrained models we show that Bayes factor does adhere to this principle and posterior predictive methods such as WAIC do not. If one of the models at hand encompasses another one, then the posterior parameter distributions of the two models may be the same. In this case, posterior predictive methods do not reflect parsimony.

Our demonstration is aimed at researchers who use analysis to state the strength of evidence from data for or against potential theoretical constraints. There are many other uses of statistics and statistical modeling that are outside this aim. Examples include researchers who model critical outcomes such as the price of a certain commodity or the perceived attractiveness of a political position. In these other endeavors—where theoretically motivated constraints are less of a concern—researchers may profit from an iterative model-building approach to minimize either within-sample or out-of-sample error. That said, when testing theories, researchers need the flexibility to specify models with overlap. And when overlap occurs, the Bayes factor is the only Bayesian method of model comparison. Another method of model comparison capable of handling overlap is the normalized maximum likelihood approach (Heck et al., 2015). For this frequentist approach, the normalizing integral is over the entire sample space, therefore accounting for model constraints on this space.

The literature on the advantages and disadvantages of different model comparison strategies is varied and voluminous, even when only focusing on Bayesian approaches. Whatever one may think about these conflicting arguments, the main point here is irrefutable. Posterior predictive methods require that to-be-compared models share no overlap for critical parameters; Bayes factors do not. Researchers who desire overlap from a substantive point of view will not profit from posterior predictive methods.

## Data Availability

No datasets were generated or analysed during the current study.

## Footnotes

1 Even though the example with the bet is a two-sample case, we show here the math for a one-sample case. The extension to two samples is just as easy yet in notation much more complicated. The figures correspond to the example and show the two-sample case.

2 For illustrative purposes, we assume independence between the three sites. If dependency between the sites was accounted for in the model the factor by which the parameter space is reduced would be smaller, and therefore, the maximum possible Bayes factor would be smaller.
